# Time period effects in work disability due to common mental disorders among young employees in Sweden—a register-based cohort study across occupational classes and employment sectors

**DOI:** 10.1093/eurpub/ckad026

**Published:** 2023-03-03

**Authors:** Ridwanul Amin, Ellenor Mittendorfer-Rutz, Emma Björkenstam, Marianna Virtanen, Magnus Helgesson, Niklas Gustafsson, Syed Rahman

**Affiliations:** Division of Insurance Medicine, Department of Clinical Neuroscience, Karolinska Institutet, Stockholm, Sweden; Division of Insurance Medicine, Department of Clinical Neuroscience, Karolinska Institutet, Stockholm, Sweden; Division of Insurance Medicine, Department of Clinical Neuroscience, Karolinska Institutet, Stockholm, Sweden; Department of Medical Sciences, Psychiatry, Uppsala University, Uppsala, Sweden; Division of Insurance Medicine, Department of Clinical Neuroscience, Karolinska Institutet, Stockholm, Sweden; School of Educational Sciences and Psychology, University of Eastern Finland, Joensuu, Finland; Division of Insurance Medicine, Department of Clinical Neuroscience, Karolinska Institutet, Stockholm, Sweden; Department of Public Health and Caring Sciences, Health Equity and Working Life, Uppsala University, Uppsala, Sweden; Division of Insurance Medicine, Department of Clinical Neuroscience, Karolinska Institutet, Stockholm, Sweden; Division of Insurance Medicine, Department of Clinical Neuroscience, Karolinska Institutet, Stockholm, Sweden

## Abstract

**Background:**

We aimed to investigate time period effects in the risk of work disability, defined as long-term sickness absence (LTSA) and disability pension (DP) due to common mental disorders (CMDs), among young employees according to employment sector (private/public) and occupational class (non-manual/manual).

**Methods:**

Three cohorts, including all employed individuals with complete information on employment sector and occupational class, aged 19–29 years and resident in Sweden on 31 December 2004, 2009 and 2014 (*n* = 573 516, 665 138 and 600 889, respectively) were followed for 4 years. Multivariate-adjusted hazard ratios (aHRs) with 95% confidence intervals (CIs) were estimated to examine the risk of LTSA and DP due to CMDs using Cox regression analyses.

**Results:**

In all cohorts, public sector employees had higher aHRs for LTSA due to CMDs than private sector employees, irrespective of occupational class, e.g. aHR, 95% CI: 1.24, 1.16–1.33 and 1.15, 1.08–1.23 among non-manual and manual workers in cohort 2004. The rates of DP due to CMDs were much lower in cohorts 2009 and 2014 than 2004 leading to uncertain risk estimates in the later cohorts. Still, public sector manual workers had a higher risk for DP due to CMDs than manual workers in the private sector in cohort 2014 than in 2004 (aHR, 95% CI: 1.54, 1.34–1.76 and 3.64, 2.14–6.18, respectively).

**Conclusions:**

Manual workers in the public sector seem to have a higher risk of work disability due to CMDs than their counterparts in the private sector calling for the need for early intervention strategies to prevent long-term work disability.

## Introduction

An increasing trend in work disability, including sickness absence (SA) and disability pension (DP) among young adults due to common mental disorders (CMDs), i.e. depressive, anxiety and stress-related mental disorders, has been reported by many EU countries, including Sweden.^1–3^ In parallel, during the previous decade, many European countries, especially Sweden, have observed a rise in the incidence of CMDs among young adults.^4–7^ Clinical characteristics of CMDs, such as early age of onset,^8^ recurrent episodes,^9^ psychosocial factors^10^^,^^11^ and high comorbidity,^12^ especially with substance use and musculoskeletal disorders,^13^ pose a potential threat to educational attainment and consequently, a negative impact on later working life.^14^

The detrimental effect of CMDs on work capacity is well established.^15–17^ Therefore, it is not unexpected that the high incidence of CMDs in young adults could result in an increased incidence of work disability. Mental disorders are now also the foremost cause for work disability in Sweden.^2^ Moreover, DP due to CMDs has increased during the past 20 years among youth in Sweden.^2^ It is therefore important to gain more knowledge on the particular risk group of young employees with work disability due to CMDs.

Previous studies identified various risk groups for developing CMDs among young employees, among them manual workers and those working in the public sector.^18^ However, few studies^19^ to date have investigated if occupational class and employment sector are related to subsequent work disability due to CMDs. A previous article from our research group showed a 29% higher risk for long-term SA (LTSA) due to CMDs among public sector vs. private sector employees.^19^ This study also reported a slightly higher risk for LTSA (11%) among manual workers vs. non-manual workers in the public sector possibly owing to the exposure to a worse psychosocial work environment and health behaviour making manual workers more vulnerable to work disability due to CMDs.^19^ However, in that study, these associations for DP due to CMDs, a permanent work disability, were not investigated. Moreover, the current literature lacks information on whether the association between employment sector and occupational class and work disability due to CMDs vary across different time periods.

Such time period effects could arise due to the mentioned temporal trends in work disability due to CMDs, meaning that some groups might be disproportionally more affected than others over time. Temporal changes in the healthcare system, in the social insurance policies, other structural changes, or even specific historical events, such as the financial crisis in 2008, could contribute to such time period effects. Particularly the major changes in the social insurance system in 2008 have remarkably reduced the incidence of permanent DP in the Swedish population aged 30+ years, but had little influence on the incidence of temporary DP among those <30 years old.^2^^,^^20^ Temporary DP continued to rise in younger individuals, especially DP due to CMDs, an increase which has started after introducing new regulations in 2003 for those <30 years.^2^ Additionally, the trend in diagnosis-specific work disability in Sweden has also varied over time, especially with a remarkable increase due to mental diagnoses since 2003.^2^ Such time period effects regarding work disability due to CMDs may differentially influence non-manual and manual workers within the public and private sectors.

As research on this topic is lacking, this study aims to investigate time period effects in the risk of work disability, defined as LTSA and DP due to CMDs, among young employees according to their employment sector (private/public) and occupational classes (non-manual/manual).

## Methods

### Study population

Three cohorts, including individuals in gainful employment, aged 19–29 years and resident in Sweden on 31 December 2004 (*n* = 731 040), 2009 (*n* = 758 846) and 2014 (*n* = 957 311) comprised the initial study population. After excluding young employees with incomplete/erroneous information on employment sector or occupational class (*n* = 157 524, 93 708 and 356 422 in the three respective cohorts), the final study population consisted of 573 516, 665 138 and 600 889 individuals for cohort 2004, 2009 and 2014, respectively.

### Data sources

We identified the study population and obtained data on age, sex, educational level, living area, family situation, emigration, occupational class and employment sector from the Longitudinal Integration Database for Health Insurance and Labour Market Studies register.^21^ Information on SA (duration, underlying diagnosis) and DP was obtained from the Micro-Data for Analysis of the Social Insurance System^22^ database. The following registers were also used: The National Patient Register: specialized healthcare^23^^,^^24^ for mental and somatic morbidities; the National Cause of Death Register:^25^ information on death. Data linkage was done by using pseudonymized unique personal identity number^26^ assigned to all individuals born or registered to be living in Sweden.

### Exposure and outcome measures

Exposure groups were categorized according to *employment sector* (private/public) and *occupational class* (non-manual/manual) with private, non-manual workers as the reference group. To avoid issues with statistical power in the analyses, the specific occupational classes were categorized into the broader non-manual/manual groups. These occupations were chosen based on the standard for Swedish occupational classification.^27^ The outcome, work disability due to CMDs, was defined as any LTSA or DP due to depressive disorders (International Classification of Diseases version 10 code, ICD-10: F32-33), phobic anxiety disorders (ICD-10: F40), other anxiety disorders (ICD-10: F41), obsessive-compulsive disorders (ICD-10: F42) and reaction to severe stress and adjustment disorders (ICD-10: F43). LTSA was defined as a sick-leave spell with >90 net SA days during the follow-up.

In Sweden, all individuals ≥16 years old are entitled to SA benefits if their work capacity is reduced by at least 25% due to disease or injury.^21^ After one qualifying day, the next 14 days of SA benefit is paid by the employer, and by the Social Insurance Agency afterwards.^21^ According to the reduction of work capacity, SA benefits can be claimed full- or part-time (i.e. 25%, 50% or 75%). All individuals 30–64 years of age with permanent impairment of work capacity due to disease or injury can be granted permanent DP.^21^ Temporary DP is granted to individuals 19–29 years of age whose work disability is expected to continue for a minimum of 1 year or who cannot complete their education due to health issues.^21^

### Covariates

A range of potential confounders, with known associations to both work-related factors and work disability due to CMDs were considered in the analyses. A directed acyclic graph (Supplementary figure 1) shows the associations among the exposure, outcome and the covariates. If not stated otherwise, all confounders were measured at baseline for each cohort. The following sociodemographic factors were included: age, sex, educational level, family situation, living area and country/region of birth (categories are shown in table 1). Health-related covariates included specific mental and somatic disorders according to ICD-10 codes shown in table 1. Diagnoses, based on specialized healthcare were measured during the 4 years before the start of the follow-up, i.e. 2001–04, 2006–09 and 2011–14, for the three respective cohorts.

**Table 1:** Sociodemographic, health-related and work-related characteristics of all employed individuals with complete information on work-related factors, aged 19–29 years, residing in Sweden in 2004, 2009 and 2014, respectively

	Cohort 2004*N* (column %)	Cohort 2009*N* (column %)	Cohort 2014*N* (column %)	Chi-squared test *P*-value
Total	573 516 (100)	665 138 (100)	600 889 (100)	
Sociodemographic characteristics^a^				
Sex				<0.0001
Female	285 483 (49.8)	328 138 (49.3)	296 421 (49.3)	
Male	288 033 (50.2)	337 000 (50.7)	304 468 (50.7)	
Age (years)				<0.0001
19–23	193 410 (33.7)	235 345 (35.4)	214 637 (35.7)	
24–29	380 106 (66.3)	429 793 (64.6)	386 252 (64.3)	
Educational level (years)				<0.0001
Compulsory school (0–9)	50 591 (8.8)	52 166 (7.8)	39 685 (6.6)	
High school (10–12)	346 558 (60.4)	399 671 (60.1)	357 095 (59.4)	
College or university (>12)	173 251 (30.2)	208 913 (31.4)	198 630 (33.1)	
Missing	3116 (0.5)	4388 (0.7)	5479 (0.9)	
Living area^b^				<0.0001
Big cities	221 820 (38.7)	270 581 (40.7)	242 885 (40.4)	
Medium-sized cities	177 469 (30.9)	238 868 (35.9)	217 356 (36.2)	
Small cities/villages	140 806 (24.6)	155 689 (23.4)	140 648 (23.4)	
Family situation				<0.0001
Married/cohabiting	109 430 (19.1)	125 987 (18.9)	92 775 (15.4)	
Not married/cohabiting^c^	464 086 (80.9)	539 151 (81.1)	508 114 (84.6)	
Country/region of birth				<0.0001
Sweden	520 883 (90.8)	593 988 (89.3)	532 277 (88.6)	
Other Nordic countries	5211 (0.9)	4161 (0.6)	3601 (0.6)	
EU25	6426 (1.1)	9584 (1.4)	10 357 (1.7)	
Rest of the world	40 983 (7.1)	57 390 (8.6)	54 654 (9.1)	
Missing	13 (0.0)	15 (0.0)	0 (0.0)	
Health-related characteristics^d^				
Any mental disorder, Yes (ICD-10^e^ codes)	15 985 (2.8)	32 941 (5.0)	60 192 (10.0)	<0.0001
Common mental disorders (F32–33, 40–43)	9354 (1.6)	21 108 (3.2)	39 585 (6.6)	
Substance use disorders (F10–19)	4209 (0.7)	7396 (1.1)	12 722 (2.1)	<0.0001
Non-affective psychosis (F20–29)	340 (0.1)	595 (0.1)	1568 (0.3)	<0.0001
Bipolar disorder (F30–31)	307 (0.1)	1324 (0.2)	5136 (0.9)	
Personality disorders (F60–69)	527 (0.1)	1529 (0.2)	4264 (0.7)	<0.0001
Attention deficit hyperactivity disorder (ADHD) (F90)	115 (0.0)	1610 (0.2)	11 252 (1.9)	<0.0001
Behavioural and emotional disorders with onset usually occurring in childhood and adolescence (Except ADHD) (F91–98)	319 (0.1)	609 (0.1)	1266 (0.2)	<0.0001
Other mental disorders (other F codes)	2221 (0.4)	2354 (0.4)	2673 (0.4)	<0.0001
Any somatic disorder (Yes)	296 374 (51.7)	304 037 (45.7)	352 350 (41.3)	<0.0001
Diabetes mellitus (E10–14)	3510 (0.6)	4287 (0.6)	4625 (0.8)	<0.0001
Epilepsy (G40)	2067 (0.4)	2364 (0.4)	2302 (0.4)	<0.0001
Asthma (J45)	5308 (0.9)	6627 (1.0)	6994 (1.2)	<0.0001
Cardiovascular disorders (I00–99)	6909 (1.2)	9790 (1.5)	9681 (1.6)	<0.0001
Neoplasm (C00-D49)	18 416 (3.2)	22 336 (3.4)	24 973 (4.2)	<0.0001
Musculoskeletal disorders (M00-99)	38 071 (6.6)	53 761 (8.1)	64 772 (10.8)	<0.0001
Other somatic disorders (other ICD-10 codes)	250 869 (43.7)	328 474 (49.4)	320 716 (53.4)	<0.0001
Work-related characteristics^a^				
Employment sector and occupational class				<0.0001
Private, non-manual	102 300 (17.8)	122 187 (18.4)	135 457 (22.5)	
Private, manual	312 488 (54.5)	401 147 (60.3)	304 850 (50.7)	
Public, non-manual	63 270 (11.0)	57 411 (8.6)	76 351 (12.7)	
Public, manual	95 458 (16.6)	84 393 (12.7)	84 231 (14.0)	

a Measured at baseline, i.e. on 31 December 2004, 2009 and 2014 for cohort 2004, 2009 and 2014, respectively.

b Big cities—Stockholm, Gothenburg and Malmö; medium-sized cities—cities with more than 90 000 inhabitants within 30 km distance from the centre of the city; small cities/villages.

c Single/divorced/widowed/separated.

d Mental and somatic disorders were measured during the 4 years before the start of follow-up, i.e. during 2001–04, 2006–09 and 2011–14, respectively, for cohort 2004, 2009 and 2014; no mental disorder and no somatic disorder groups are not presented.

e International Classification of Diseases version 10.

### Statistical analysis

Differences in the distribution of sociodemographic and health-related characteristics among the cohorts were estimated by the Chi-squared test. Cramér’s V was used to test for multicollinearity among the nominal covariates. Crude and multivariate hazard ratios (HRs and aHRs, respectively) of work disability due to CMDs were estimated by Cox regression models. The assumption of proportional hazard was confirmed by plotting log-minus-log Kaplan–Meier survival curves. Each cohort was followed up for 4 years (i.e. from 1 January 2005, 2010 and 2015 to 31 December 2008, 2013 and 2018, respectively, for each cohort) regarding the outcome measures. Censoring was done in the event of emigration, death and end of follow-up, whichever occurred first. For the outcome of LTSA due to CMDs, an additional censoring event was any DP. Individuals with long-term work disability at baseline were excluded, i.e. for the analyses of LTSA as the outcome, individuals with ongoing LTSA or DP (*n* = 19 899, 12 779 and 18 309 in cohort 2004, 2009 and 2014, respectively), and for the analysis of DP as the outcome, individuals with ongoing DP at baseline (*n* = 1208, 1593 and 1956 in cohort 2004, 2009 and 2014, respectively). All analyses were performed using SAS 9.4.

### Ethical approval

The project was approved by the Regional Ethical Review Board, Karolinska Institutet, Stockholm, Sweden (dnr: 2007/762-31).

## Results

The distribution of the sociodemographic factors was similar across all three cohorts (table 1). There was a chronological increase in the proportion of individuals with a mental disorder at baseline across the cohorts (3%, 5% and 10% in cohort 2004, 2009 and 2014, respectively). In all three cohorts, most individuals worked in the private sector (about 73% in the cohort 2004 and 2014, and 79% in the cohort 2009), and as manual workers (around 73% across the cohorts).

### LTSA due to CMDs

**Figure 1:**
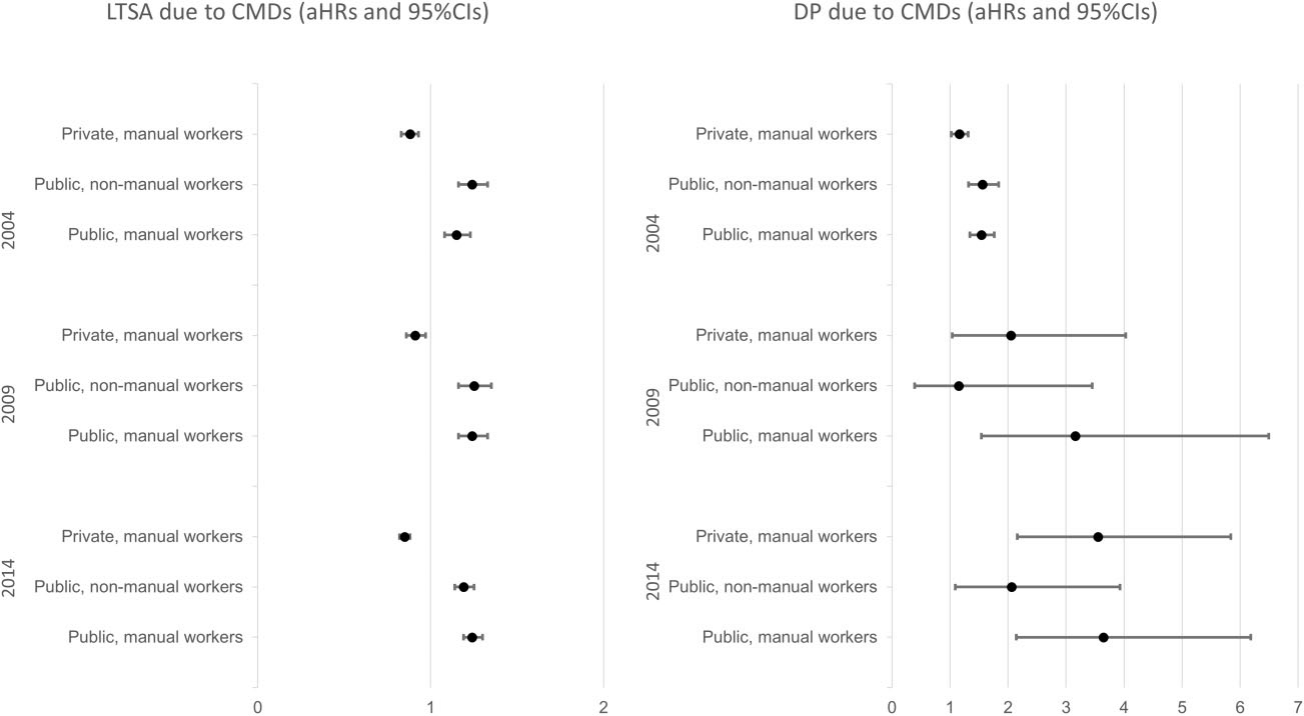
Multivariate-adjusted hazard ratios (aHRs) with 95% confidence intervals (CI) for long-term sickness absence (LTSA) and disability pension (DP) due to common mental disorders (CMDs), in employed individuals aged 19–29 years residing in Sweden in 2004, 2009 and 2014; the reference group is private, non-manual workers

The rates of LTSA due to CMDs were much higher in the cohort 2014 than in the earlier cohorts. Generally, public sector employees, among both occupational classes, had a statistically significant higher risk (HRs and aHRs) of LTSA due to CMDs than private sector employees across all three cohorts (table 2 and figure 1). Within the private sector, manual workers had a slightly lower risk (aHRs) of LTSA due to CMDs than non-manual workers (table 2 and figure 1). These differences were more prominent in cohort 2014 (17%) than in the earlier cohorts (12% and 8% in cohort 2004 and 2009, respectively).

**Table 2:** Crude and multivariate-adjusted hazard ratios (HRs and aHRs, respectively) with 95% confidence intervals (CI) for long-term sickness absence (LTSA) due to common mental disorders, in employed individuals aged 19–29 years residing in Sweden in 2004, 2009 and 2014 (cohort 2004, 2009 and 2014, respectively) who had no ongoing LTSA or disability pension at baseline

Employment sector and occupational class	*n* (rate per 100 000 person-years)	Crude model HR (95%CI)	Adjusted model^a^ aHR (95%CI)
Cohort 2004			
Private, non-manual	1869 (470.0)	1 (Ref.)	1 (Ref.)
Private, manual	5020 (416.0)	0.88 (0.84–0.93)	0.88 (0.83–0.93)
Public, non-manual	1523 (625.0)	1.33 (1.25–1.43)	1.24 (1.16–1.33)
Public, manual	2799 (774.3)	1.65 (1.56–1.75)	1.15 (1.08–1.23)
Cohort 2009			
Private, non-manual	1766 (367.0)	1 (Ref.)	1 (Ref.)
Private, manual	5530 (352.4)	0.95 (0.91–1.01)	0.91 (0.86–0.97)
Public, non-manual	1183 (525.6)	1.43 (1.33–1.54)	1.25 (1.16–1.35)
Public, manual	2224 (678.8)	1.85 (1.74–1.97)	1.24 (1.16–1.33)
Cohort 2014			
Private, non-manual	5134 (976.6)	1 (Ref.)	1 (Ref.)
Private, manual	9309 (790.9)	0.80 (0.78–0.83)	0.85 (0.82–0.88)
Public, non-manual	3773 (1396.9)	1.32 (1.26–1.37)	1.19 (1.14–1.25)
Public, manual	5139 (1583.8)	1.63 (1.57–1.70)	1.24 (1.19–1.30)

a Adjusted for: age, sex, educational level, living area, family situation, country/region of birth, mental disorder at baseline (common mental disorders, substance abuse, non-affective psychosis, bipolar disorder, personality disorder, ADHD, behavioural and emotional disorders other than ADHD, other mental disorders) and somatic disorder at baseline (diabetes mellitus, epilepsy, asthma, cardiovascular disorders, neoplasm, musculoskeletal disorders, other somatic disorders).

### DP due to CMDs

The rates of DP due to CMDs were considerably higher in the cohort 2004 compared with the later cohorts (table 3). These rates were highest among manual workers in the public sector across all three cohorts (80, 13 and 23 per 100 000 in cohort 2004, 2009 and 2014, respectively) than the other groups (table 3). In the cohort 2004, public sector employees, irrespective of their occupational class, had a statistically significant higher risk (aHR, 95% CI: 1.56, 1.32–1.84 and 1.54, 1.34–1.76 for non-manual and manual workers in the public sector, respectively) of DP due to CMDs than private sector employees. Such differences between the sectors were not apparent in the later cohorts where the corresponding analytical models lacked statistical power (table 3 and figure 1). Still, public sector manual workers had a higher risk for DP due to CMDs than manual workers in the private sector in cohort 2014 than in 2004 (aHR, 95% CI: 1.54, 1.34–1.76 and 3.64, 2.14–6.18, respectively).

**Table 3:** Crude and multivariate-adjusted hazard ratios (HRs and aHRs, respectively) with 95% confidence intervals (CI) for disability pension (DP) due to common mental disorders, in employed individuals aged 19–29 years residing in Sweden in 2004, 2009 and 2014 (cohort 2004, 2009 and 2014, respectively) who had no ongoing DP at baseline

Employment sector and occupational class	*n* (rate per 100 000 person-years)	Crude model HR (95% CI)	Adjusted model^a^ aHR (95% CI)
Cohort 2004			
Private, non-manual	115 (28.2)	1 (Ref.)	1 (Ref.)
Private, manual	485 (38.9)	1.47 (1.31–1.65)	1.16 (1.02–1.31)
Public, non-manual	96 (38.0)	1.42 (1.22–1.66)	1.56 (1.32–1.84)
Public, manual	304 (79.9)	2.70 (2.38–3.07)	1.54 (1.34–1.76)
Cohort 2009			
Private, non-manual	10 (2.1)	1 (Ref.)	1 (Ref.)
Private, manual	109 (6.8)	3.31 (1.73–6.32)	2.05 (1.04–4.03)
Public, non-manual	<10^b^	1.06 (0.36–3.10)	1.15 (0.39–3.45)
Public, manual	44 (13.1)	6.34 (3.19–12.60)	3.16 (1.54–6.49)
Cohort 2014			
Private, non-manual	18 (3.3)	1 (Ref.)	1 (Ref.)
Private, manual	193 (15.9)	4.76 (2.94–7.71)	3.55 (2.16–5.84)
Public, non-manual	21 (6.9)	2.07 (1.10–3.88)	2.06 (1.09–3.93)
Public, manual	78 (23.2)	6.96 (4.17–11.62)	3.64 (2.14–6.18)

a Adjusted for: age, sex, educational level, living area, family situation and country/region of birth, mental disorder at baseline (common mental disorders, substance abuse, non-affective psychosis, bipolar disorder, personality disorder, ADHD, behavioural and emotional disorders other than ADHD, other mental disorders) and somatic disorder at baseline (diabetes mellitus, epilepsy, asthma, cardiovascular disorders, neoplasm, musculoskeletal disorders, other somatic disorders).

b For ethical reasons, if the number of DP is <10, it is not reported.

### Sensitivity analysis

To disentangle cohort and period effects, a sensitivity analysis was carried out for those aged 19–23 years at baseline by making the study populations of the individual time period cohorts mutually exclusive. The results from these analyses closely resembled our main findings for all three cohorts (Supplementary tables S1 and S2). The similarity between the results from the main and the sensitivity analysis, therefore, suggests the identified differences are more due to period effects than for cohort effects.

## Discussion

### Main findings

This population-based cohort study investigated the time period effects in work disability due to CMDs among young employees across employment sectors and occupational classes. Among both employment sectors and occupational classes, rates of LTSA due to CMDs were almost twice as high in the cohort 2014 than in the earlier cohorts (2004 and 2009). Public sector employees had a higher relative risk for LTSA due to CMDs than private sector employees, irrespective of their occupational classes, and these associations were similar across all cohorts. The rate of DP due to CMDs was considerably higher in the cohort 2004 than the later ones. In all three cohorts, rates of DP were higher among the manual workers in the public sector than the other groups. The relative risk of DP due to CMDs in the cohort 2004 was higher among public sector employees, regardless of their occupational classes, than private sector employees. Although the results from the cohort 2004 were less comparable due to lack of statistical power in the later cohorts, it still showed that, public sector manual workers had a somewhat higher risk for DP due to CMDs than manual workers in the private sector in cohort 2014 than in 2004.

### Employment sector, occupational class and LTSA due to CMDs

The rates of LTSA due to CMDs among the different employment sector and occupational class groups were nearly two times higher in the cohort 2014 than in the earlier cohorts. Our finding is coherent with the Swedish national trend, showing an increase in the LTSA following a few years after major insurance policy changes in 2008.^2^ However, the policy changes alone might not only explain such a big difference among the cohorts. It is also likely to be driven by other factors such as the increasing trend of CMDs in the younger population since early 2000^4^^,^^6^^,^^28^ and the healthcare system not meeting the demand of such increasing trend.^1^^,^^5^ Our study also suggests that the increasing trend was observed not only for CMDs but also for other mental diagnoses, with more than three times higher prevalence in cohort 2014 than in cohort 2004. These findings may partly be explained by the fact that the coverage of the specialized outpatient register in Sweden has increased markedly between 2004 and 2014.^29^

Across all three cohorts, rates and relative risk estimates of LTSA due to CMDs were higher among employees in the public sector than the private sector. In a previous study from our research group, with the same study population as cohort 2009 in the present study but a longer follow-up period (7 years), similar findings regarding LTSA due to CMDs among young employees in Sweden were reported.^19^ Regarding the occupational classes within the different sectors, we found a lower risk for LTSA due to CMDs among manual workers than non-manual workers in the private sector. The novelty of this result requires further exploration to understand the underlying mechanisms. For example, the nature of manual work in the private sector can be more favourable for better recovery and early return to work. Moreover, the rehabilitation programmes in the private sector could be more effective to prevent LTSA. Furthermore, the fear of job loss may lead to an early return to work. Additionally, there could be selection processes involved; the high demands on high productivity in the private sector may lead to retaining only the ‘healthier’ manual workers in this sector. These mechanisms regarding the occupational classes may differ between the private and the public sector and should be investigated in future studies. Additionally, the distribution of the occupational branches within the employment sectors may also contribute to these differences in work disability. Public sector employees are more often from the healthcare branch than private sector employees (Supplementary table S3) and these professions are emotionally more demanding, have limited work–time control and often include shift work during nights.^19^

Within the public sector, the risk of LTSA due to CMDs did not vary significantly according to the occupational classes. These results contradict the findings from two previous studies where manual workers in the Finnish public sector had a higher risk of depression-related work disability than non-manual workers.^30^^,^^31^ Recent literature on diagnosis-specific SA trends among manual and non-manual workers reports that SA due to mental diagnoses among manual workers has undergone a substantial decrease over the past years.^32^^,^^33^ Therefore, the discrepancies between our results and the Finnish studies might have been due to the differences in the definition of SA itself, i.e. duration, or the considered underlying cause for SA. We have also found that the crude risk of LTSA due to CMDs was higher among manual workers than non-manual workers in the public sector. Interestingly, these crude differences attenuated after adjusting for the potential confounding factors, particularly the educational level. These results may highlight the importance of providing additional support for manual workers in the public sector with lower educational level regarding their return to work following SA due to CMDs.

### Employment sector, occupational class and DP due to CMDs

The rates of DP were much higher among the different groups in the cohort 2004 than in the same groups in the later cohorts. This finding is not surprising given that much stricter regulations regarding receipt of DP were integrated into the Swedish Insurance Policy in 2008, which most likely have affected the later cohorts.^2^ A nearly 6-fold decrease in the incidence of DP due to CMDs following regulation changes was previously reported.^20^

Public sector employees in the cohort 2004 had a higher risk of DP due to CMDs than private sector employees. Similar differences regarding the employment sector were not observed in the later cohorts, primarily due to a much lower number of DPs leading to uncertain risk estimates. A lack of previous studies investigating the risk of DP according to the employment sector limited the comparability of our results. Although stress levels are considered to be generally higher in the public sector,^19^ a previous study in Australia did not find differences between the public and the private sectors regarding psychological distress and job satisfaction.^34^ Until replication in future studies, our results should be interpreted with caution. Future studies should investigate to what extent psychosocial, and work-related factors differ between sectors of employment and how that may affect the risk of DP among employees.

Within each time period cohort, the rates of DP due to CMDs were higher among manual workers in the public sector than in any other group. In addition, although not always statistically significant, we found a higher relative risk (aHR) of DP due to CMDs among manual workers vs. non-manual workers within both private and public sectors in all three cohorts except for the public sector employees in cohort 2004 (no difference by occupational class). These results are partially comparable with a previous study in Norway which reported a 2-fold higher risk (HR) of receipt of DP among manual workers compared with non-manual workers although no stratification by employment sector was carried out and the outcome was all-cause DP.^35^

### Work disability due to CMDs across the time period cohorts

During the study period, among all newly diagnosed SA in Sweden, SA due to mental disorders increased steadily from 75% in 2005 to around 86% in 2018.^2^ However, this change in overall trend did not seem to differentially affect our exposure groups, i.e. the association of LTSA due to CMDs across employment sectors and occupational classes did not differ significantly across the time period cohorts. On the other hand, there was limited comparability across the cohorts regarding the association between DP due to CMDs, and employment sectors and occupational classes due to lack of statistical power in the later cohorts. While the directions of the associations were similar across all three cohorts, i.e. a higher relative risk among public vs. private sector employees and manual vs. non-manual workers, these associations were more prominent in the cohort 2004 than the other cohorts. The differences in the findings were primarily due to a much lower incidence of DP among the different exposure groups in the cohorts 2009 and 2014. Even after considering the limited comparability across cohorts due to lower statistical power in the later cohorts, our results still showed that public sector manual workers had somewhat higher risk of DP due to CMDs in cohort 2014 than in 2004. These findings highlight the increasing disparity regarding long-term work disability for young manual workers in the public sector. This public health concern needs to be addressed by interventions that improve conditions in the working environment of the manual workers in the public sector and, therefore, can contribute to prevent long-term work disability due to CMDs among this vulnerable group.

### Strengths and limitations

The main strengths of our study include the population-based longitudinal design, use of nationwide registers with good validity and completeness,^21^^,^^23^^,^^25^ and minimum risk with loss to follow-up. Additionally, we could include the entire population of employed youth, which allowed us to perform several stratified analyses, e.g. by employment sector and occupational class, while controlling for several potential confounders. However, our results should be interpreted considering several limitations. First, differential exclusion due to missing data on work-related factors may have introduced selection bias in our study. These variations reflect changes in coding practices by Statistics Sweden over the years regarding these variables.^27^ However, our sensitivity analyses (Supplementary table S4) revealed no differences between the excluded groups across the cohorts regarding the risk of LTSA or DP. Moreover, the sociodemographic and health-related characteristics within individual cohorts were quite similar between the excluded individuals vs. the included study population (Supplementary table S5). Therefore, selection bias does not seem to have affected our results. Second, as we have extracted the medical factors from the healthcare registers, we may have missed less severe morbidity cases, resulting in potential residual confounding. Residual confounding may also arise from the lack of information on lifestyle and work environmental factors in the registers which were not accounted for in the studied associations.

## Conclusion

Manual workers in the public sector seem to have a higher risk of work disability due to CMDs than their private sector peers. For this vulnerable group, the risk for LTSA due to CMDs was stable across all cohorts despite strong differences in the rates of such work disability over time. Therefore, manual workers in the public sector need specific attention with early intervention strategies in order to prevent long-term work disability due to CMDs among them.

## Supplementary Material

ckad026_Supplementary_Data

## Data Availability

The data used in this study cannot be made publicly available due to privacy regulations. According to the General Data Protection Regulation, the Swedish law SFS 2018:218, the Swedish Data Protection Act, the Swedish Ethical Review Act and the Public Access to Information and Secrecy Act. These types of sensitive data can only be made available for specific purposes, including research that meets the criteria for access to such sensitive and confidential data as determined by a legal review. Readers may contact Professor Kristina Alexanderson (kristina.alexanderson@ki.se) regarding the data access. Key points Public sector employees had a higher relative risk for long-term sickness absence due to common mental disorders than private sector employees, irrespective of their occupational classes. Manual workers in the public sector seem to have a higher risk of work disability due to CMDs than non-manual workers in the private sector with a tendency to an increased risk over time for permanent work disability. Manual workers in the public sector should be the focus of early intervention strategies to prevent long-term work disability.
