# Supplementary material for: Time period effects in work disability due to common mental disorders among young employees in Sweden—a register-based cohort study across occupational classes and employment sectors
Source: Eur J Public Health. 2023 Mar 3;33(2):272–8. doi: 10.1093/eurpub/ckad026 (PMC10066471; doi:10.1093/eurpub/ckad026)

**Supplementary Table S1.** Crude and multivariate-adjusted hazard ratios (HRs and aHRs, respectively) with 95% confidence intervals (CI) for long-tern sickness absence (LTSA) due to common mental disorders (CMDs), in employed individuals *aged 19-23 years* residing in Sweden in 2004, 2009 and 2014 (cohort 2004, 2009 and 2014, respectively) who had no ongoing LTSA or disability pension at baseline.

| Employment sector and occupational class | n (rate per 100 000 person-years) | Crude Model HR (95%CI) | Adjusted Model^1^ aHR (95%CI) |
| --- | --- | --- | --- |
| Cohort 2004 |  |  |  |
| Private, non-manual | 202 (414) | 1 (Ref.) | 1 (Ref.) |
| Private, manual | 1779 (341) | 0.82 (0.71 – 0.95) | 0.79 (0.68 - 0.91) |
| Public, non-manual | 86 (417) | 1.00 (0.78 – 1.29) | 1.09 (0.82 - 1.36) |
| Public, manual | 930 (564) | 1.36 (1.17 – 1.58) | 0.99 (0.85 - 1.16) |
| Cohort 2009 |  |  |  |
| Private, non-manual | 220 (294) | 1 (Ref.) | 1 (Ref.) |
| Private, manual | 1840 (267) | 0.90 (0.79 - 1.04) | 0.87 (0.75 - 1.00) |
| Public, non-manual | 69 (373) | 1.27 (0.97 - 1.66) | 1.33 (1.01 - 1.74) |
| Public, manual | 704 (483) | 1.64 (1.41 - 1.91) | 1.23 (1.06 - 1.44) |
| Cohort 2014 |  |  |  |
| Private, non-manual | 913 (828) | 1 (Ref.) | 1 (Ref.) |
| Private, manual | 3150 (598) | 0.72 (0.67 - 0.77) | 0.72 (0.67 - 0.77) |
| Public, non-manual | 414(871) | 1.05 (0.94 - 1.18) | 1.07 (0.95 - 1.20) |
| Public, manual | 1828 (1175) | 1.42 (1.31 - 1.54) | 1.07 (0.99 - 1.16) |

^1^Adjusted for: sex, educational level, living area, family situation, and country/region of birth, mental disorder at baseline (common mental disorders, substance abuse, non-affective psychosis, bipolar disorder, personality disorder, ADHD, behavioural and emotional disorders other than ADHD, other mental disorders), somatic disorder at baseline (diabetes mellitus, epilepsy, asthma, cardiovascular disorders, neoplasm, musculoskeletal disorders, other somatic disorders)

**Supplementary Table S2.** Crude and multivariate-adjusted hazard ratios (HRs and aHRs, respectively) with 95% confidence intervals (CI) for disability pension (DP) due to common mental disorders (CMDs), in employed individuals *aged 19-23 years* residing in Sweden in 2004, 2009 and 2014 (cohort 2004, 2009 and 2014, respectively) who had no ongoing DP at baseline.

| Employment sector and occupational class | n (rate per 100 000 person-years) | Crude Model HR (95%CI) | Adjusted Model^1^ aHR (95%CI) |
| --- | --- | --- | --- |
| Cohort 2004 |  |  |  |
| Private, non-manual | 27 (54) | 1 (Ref.) | 1 (Ref.) |
| Private, manual | 146 (27) | 0.71 (0.55 – 0.93) | 1.03 (0.91 - 1.17) |
| Public, non-manual | <10^2^ | 0.95 (0.59 – 1.52) | 1.53 (1.30 - 1.80) |
| Public, manual | 89 (53) | 1.34 (1.02 – 1.78) | 1.36 (1.19 - 1.56) |
| Cohort 2009 |  |  |  |
| Private, non-manual | <10^2^ | 1 (Ref.) | 1 (Ref.) |
| Private, manual | 47 (7) | 1.69 (0.53 - 5.43) | 1.37 (0.43 - 4.42) |
| Public, non-manual | <10^2^ | 2.69 (0.45 - 16.07) | 3.45 (0.57 - 20.97) |
| Public, manual | 22 (15) | 3.74 (1.12 - 12.48) | 2.69 (0.79 - 9.14) |
| Cohort 2014 |  |  |  |
| Private, non-manual | <10^2^ | 1 (Ref.) | 1 (Ref.) |
| Private, manual | 116 (22) | 6.07 (2.24 - 16.45) | 5.90 (2.16 - 16.08) |
| Public, non-manual | <10^2^ | 2.34 (0.59 - 9.35) | 2.58 (0.64 - 10.41) |
| Public, manual | 36 (23) | 6.41 (2.28 - 18.02) | 3.66 (1.29 - 10.42) |

^1^Adjusted for: sex, educational level, living area, family situation, and country/region of birth, mental disorder at baseline (common mental disorders, substance abuse, non-affective psychosis, bipolar disorder, personality disorder, ADHD, behavioural and emotional disorders other than ADHD, other mental disorders), somatic disorder at baseline (diabetes mellitus, epilepsy, asthma, cardiovascular disorders, neoplasm, musculoskeletal disorders, other somatic disorders)
^2^For ethical reasons, if the number of DP is <10, it is not reported

**Supplementary Table S3.** Distribution of the occupational branches across employment sectors and occupational classes in the three cohorts.

| **Employment sector and occupational class** | **All n, column %** | **Agriculture n, row %** | **Commerce and Transport n, row %** | **Construction n, row %** | **Education and research n, row %** | **Financial and enterprise n, row %** | **Healthcare n, row %** | **Industry n, row %** | **Others n, row %** |
| --- | --- | --- | --- | --- | --- | --- | --- | --- | --- |
| **Cohort 2004** |  |  |  |  |  |  |  |  |  |
| Private, non-manual | 102,300 (17.8) | 457 (0.4) | 22,750 (22.2) | 2,481 (2.4) | 5,700 (5.6) | 37,841 (37.0) | 3,981 (3.9) | 18,371 (18.0) | 10,719 (10.5) |
| Private, manual | 312,488 (54.5) | 5,500 (1.8) | 113,135 (36.2) | 26,115 (8.4) | 4,036 (1.3) | 34,278 (11.0) | 12.761 (4.1) | 69,761 (22.3) | 46,902 (15.0) |
| Public, non-manual | 63,270 (11.0) | 37 (0.1) | 405 (0.6) | 343 (0.5) | 26,626 (42.1) | 764 (1.2) | 18,223 (28.8) | 9 (0.01) | 16,863 (26.6) |
| Public, manual | 95,458 (16.6) | 92 (0.1) | 420 (0.4) | 833 (0.9) | 17,691 (18.5) | 1,013 (1.1) | 62,505 (65.5) | 6 (0.01) | 12,898 (13.5) |
| **Cohort 2009** |  |  |  |  |  |  |  |  |  |
| Private, non-manual | 122,187 (18.4) | 573 (0.5) | 24,247 (19.8) | 3,925 (3.2) | 5,740 (4.7) | 12,028 (9.8) | 4,468 (3.7) | 15,513 (12.7) | 55,693 (45.6) |
| Private, manual | 401,147 (60.3) | 6,802 (1.7) | 180,930 (45.1) | 47,034 (11.7) | 5,833 (1.4) | 5,910 (1.5) | 22,327 (5.6) | 59,304 (14.8) | 73,007 (18.2) |
| Public, non-manual | 57,411 (8.6) | 10 (0.02) | 288 (0.5) | 260 (0.4) | 19,364 (33.7) | 243 (0.4) | 19,383 (33.8) | 51 (0.1) | 17,812 (31.0) |
| Public, manual | 84,393 (12.7) | 22 (0.03) | 723 (0.9) | 758 (0.9) | 15,583 (18.5) | 206 (0.2) | 55,011 (65.2) | 188 (0.2) | 11,902 (14.1) |
| **Cohort 2014** |  |  |  |  |  |  |  |  |  |
| Private, non-manual | 135,457 (22.5) | 326 (0.2) | 32,358 (23.9) | 4,427 (3.3) | 6,385 (4.7) | 12,870 (9.5) | 5,451 (4.0) | 13,227 (9.8) | 60,413 (44.6) |
| Private, manual | 304,850 (50.7) | 4,001 (1.3) | 135,880 (44.6) | 41,808 (13.7) | 5,055 (1.7) | 2,397 (0.8) | 23,182 (7.6) | 48,012 (15.7) | 44,515 (14.6) |
| Public, non-manual | 76,351 (12.7) | 14 (0.02) | 379 (0.5) | 259 (0.3) | 23,363 (30.6) | 344 (0.4) | 23,783 (31.1) | 86 (0.1) | 28123 (36.8) |
| Public, manual | 84,231 (14.0) | 7 (0.01) | 345 (0.4) | 641 (0.8) | 20,103 (23.9) | 266 (0.3) | 52,308 (62.1) | 219 (0.3) | 10,342 (12.3) |

**Supplementary Table S4**. Crude and multivariate-adjusted hazard ratios (HRs and aHRs, respectively) with 95% confidence intervals (CI) for long-term sickness absence (LTSA) and disability pension (DP) due to common mental disorders (CMDs) among the excluded individuals, compared with the included individuals, in the three time period cohorts.

| **LTSA** | **Crude Model HR (95%CI)** | **Adjusted Model^1^ aHR (95%CI)** |
| --- | --- | --- |
| Cohort 2004 | 0.74 (0.71 – 0.78) | 0.79 (0.76 – 0.83) |
| Cohort 2009 | 0.61 (0.57 – 0.65) | 0.64 (0.60 – 0.69) |
| Cohort 2014 | 0.86 (0.84 – 0.88) | 0.86 (0.84 – 0.88) |
| **DP** |  |  |
| Cohort 2004 | 1.40 (1.25 – 1.58) | 1.39 (1.24 – 1.57) |
| Cohort 2009 | 2.60 (1.94 – 3.49) | 2.16 (1.61 – 2.91) |
| Cohort 2014 | 2.43 (2.10 – 2.81) | 2.28 (1.97 – 2.63) |

^1^Adjusted for: sex, educational level, living area, family situation, and country/region of birth, mental disorder at baseline (common mental disorders, substance abuse, non-affective psychosis, bipolar disorder, personality disorder, ADHD, behavioural and emotional disorders other than ADHD, other mental disorders), somatic disorder at baseline (diabetes mellitus, epilepsy, asthma, cardiovascular disorders, neoplasm, musculoskeletal disorders, other somatic disorders)

**Supplementary Table S5.** Sociodemographic and health-related characteristics of the excluded and included individuals in the three time period cohorts.

|  | Cohort 2004 | | Cohort 2009 | | Cohort 2014 | |
| --- | --- | --- | --- | --- | --- | --- |
|  | **Excluded** | **Included** | **Excluded** | **Included** | **Excluded** | **Included** |
|  | **N (column %)** |  | **N (column %)** |  | **N (column %)** |  |
| Total (row %) | 157,524 (21.5) | 573,516 (78.5) | 93,708 (12.4) | 665,138 (87.6) | 356,422 (37.2) | 600,889 (62.8) |
| Sociodemographic characteristics^*^ |  |  |  |  |  |  |
| *Sex* |  |  |  |  |  |  |
| Female | 94,250 (59.8) | 288,033 (50.2) | 56,079 (59.8) | 337,000 (50.7) | 185,724 (52.1) | 304,468 (50.7) |
| Male | 63,274 (40.2) | 285,483 (49.8) | 37,629 (40.2) | 328,138 (49.3) | 170,698 (47.9) | 296,421 (49.3) |
| *Age (years)* |  |  |  |  |  |  |
| 19-23 | 81,930 (52) | 193,410 (33.7) | 67,549 (72.1) | 235,346 (35.4) | 152,807 (42.9) | 214,637 (35.7) |
| 24-29 | 75,594 (48) | 380,106 (66.3) | 26,159 (27.9) | 429,793 (64.6) | 203,615 (57.1) | 386,252 (64.3) |
| *Educational level (years)* |  |  |  |  |  |  |
| Compulsory school (0-9) | 18,559 (11.8) | 50,591 (8.8) | 11,591 (12.4) | 52,166 (7.8) | 35,666 (10) | 39,685 (6.6) |
| High school (10-12) | 99,264 (63) | 346,558 (60.4) | 62,567 (66.8) | 399,671 (60.1) | 218,510 (61.3) | 357,095 (59.4) |
| College or university (>12) | 37,542 (23.8) | 173,251 (30.2) | 16,943 (18.1) | 208,913 (31.4) | 96,828 (27.2) | 198,630 (33.1) |
| Missing | 2159 (1.4) | 3116 (0.5) | 2607 (2.8) | 4388 (0.7) | 5418 (1.5) | 5479 (0.9) |
| *Living Area^2^* |  |  |  |  |  |  |
| Big cities | 62,362 (39.6) | 221,820 (38.7) | 37,754 (40.3) | 270,581 (40.7) | 150,451 (42.2) | 242,885 (40.4) |
| Medium-sized cities | 56,654 (36) | 210,890 (36.8) | 32,557 (34.7) | 238,868 (35.9) | 123,609 (34.7) | 217,356 (36.2) |
| Small cities/villages | 38,508 (24.4) | 140,806 (24.6) | 23,397 (25) | 155,689 (23.4) | 82,362 (23.1) | 140,648 (23.4) |
| *Family situation* |  |  |  |  |  |  |
| Married/cohabiting | 22,186 (14.1) | 109,430 (19.1) | 8619 (9.2) | 125,987 (18.9) | 62,378 (17.5) | 92,775 (15.4) |
| Not married/cohabiting | 135,338 (85.9) | 464,086 (80.9) | 85,089 (90.8) | 539,151 (81.1) | 294,044 (82.5) | 508,114 (84.6) |
| *Country/region of birth* |  |  |  |  |  |  |
| Sweden | 139,019 (88.3) | 520,883 (90.8) | 78,079 (83.3) | 593,998 (89.3) | 308,667 (86.6) | 532,277 (88,6) |
| Other Nordic countries | 1607 (1) | 5211 (0.9) | 852 (0.9) | 4161 (0.6) | 2234 (0.6) | 3601 (0,6) |
| EU25 | 2467 (1.6) | 6426 (1.1) | 3020 (3.2) | 9584 (1.4) | 7887 (2.2) | 10,357 (1,7) |
| Rest of the world | 14,431 (9.2) | 40,996 (7.1) | 11,757 (12.5) | 57,405 (8.6) | 37,634 (10.6) | 54,654 (9,1) |

**Supplementary Table S5 (continued).** Sociodemographic and health-related characteristics of the excluded and included individuals in the three time period cohorts.

| Health-related characteristics^4^ |  |  |  |  |  |  |
| --- | --- | --- | --- | --- | --- | --- |
| *Any mental disorder (Yes)* | 5210 (3,3) | 15,985 (2,8) | 5223 (5,6) | 32,941 (5) | 49,125 (13,8) | 60,192 (10) |
| Substance use disorders | 1585 (1) | 4209 (0,7) | 1404 (1,5) | 7396 (1,1) | 11,451 (3,2) | 12,722 (2,1) |
| Non-affective psychosis | 160 (0,1) | 340 (0,1) | 120 (0,1) | 595 (0,1) | 1776 (0,5) | 1568 (0,3) |
| Bipolar disorder | 82 (0,1) | 307 (0,1) | 152 (0,2) | 1324 (0,2) | 5017 (1,4) | 5136 (0,9) |
| Personality disorders | 189 (0,1) | 527 (0,1) | 196 (0,2) | 1529 (0,2) | 4050 (1,1) | 4264 (0,7) |
| Attention deficit hyperactivity disorder (ADHD) | 120 (0,1) | 319 (0,1) | 147 (0,2) | 609 (0,1) | 1073 (0,3) | 1266 (0,2) |
| Behavioral and emotional disorders with onset usually occurring in childhood and adolescence (Except ADHD) | 83 (0,1) | 115 (0) | 468 (0,5) | 1610 (0,2) | 10,352 (2,9) | 11,252 (1,9) |
| Other mental disorders | 649 (0,4) | 2221 (0,4) | 428 (0,5) | 2354 (0,4) | 1808 (0,5) | 2673 (0,4) |
| *Any somatic disorder (Yes)* | 75,022 (47,6) | 277,142 (48,3) | 48,248 (51,5) | 359,112 (54) | 215,300 (60,4) | 352,350 (58,6) |
| Diabetes mellitus | 867 (0,6) | 3510 (0,6) | 529 (0,6) | 4287 (0,6) | 2659 (0,7) | 4625 (0,8) |
| Epilepsy | 555 (0,4) | 2067 (0,4) | 304 (0,3) | 2364 (0,4) | 10,528 (3) | 12,203 (2) |
| Asthma | 1626 (1) | 5308 (0,9) | 1287 (1,4) | 6627 (1) | 4441 (1,2) | 6994 (1,2) |
| Cardiovascular disorders | 1852 (1,2) | 6909 (1,2) | 1160 (1,2) | 9790 (1,5) | 6120 (1,7) | 9618 (1,6) |
| Neoplasm | 4560 (2,9) | 18,416 (3,2) | 2615 (2,8) | 22,336 (3,4) | 14,283 (4) | 24,973 (4,2) |
| Musculoskeletal disorders | 10,289 (6,5) | 38,071 (6,6) | 7266 (7,8) | 53,761 (8,1) | 39,757 (11,2) | 64,772 (10,8) |
| Other somatic disorders | 68,085 (43,2) | 250,869 (43,7) | 44,138 (47,1) | 328,474 (49,4) | 197,559 (55,4) | 320,716 (53,4) |

^1^ Measured at baseline, i.e., on 31 December 2004, 2009 and 2014 for cohort 2004, 2009 and 2014, respectively

^2^ Big cities—Stockholm, Gothenburg and, Malmö; medium-sized cities—cities with more than 90 000 inhabitants within 30 km distance from the center of the city; small cities/villages

^3^ Single/divorced/widowed/separated

^4^ Mental and somatic disorders were measured during the four years before the start of follow-up, i.e., during 2001-2004, 2006-2009, and 2011-2014, respectively for cohort 2004, 2009 and 2014; no mental disorder and no somatic disorder groups are not presented.

**Supplementary Figure 1.** Directed acyclic graph of the associations among the exposure, outcome and the covariates.


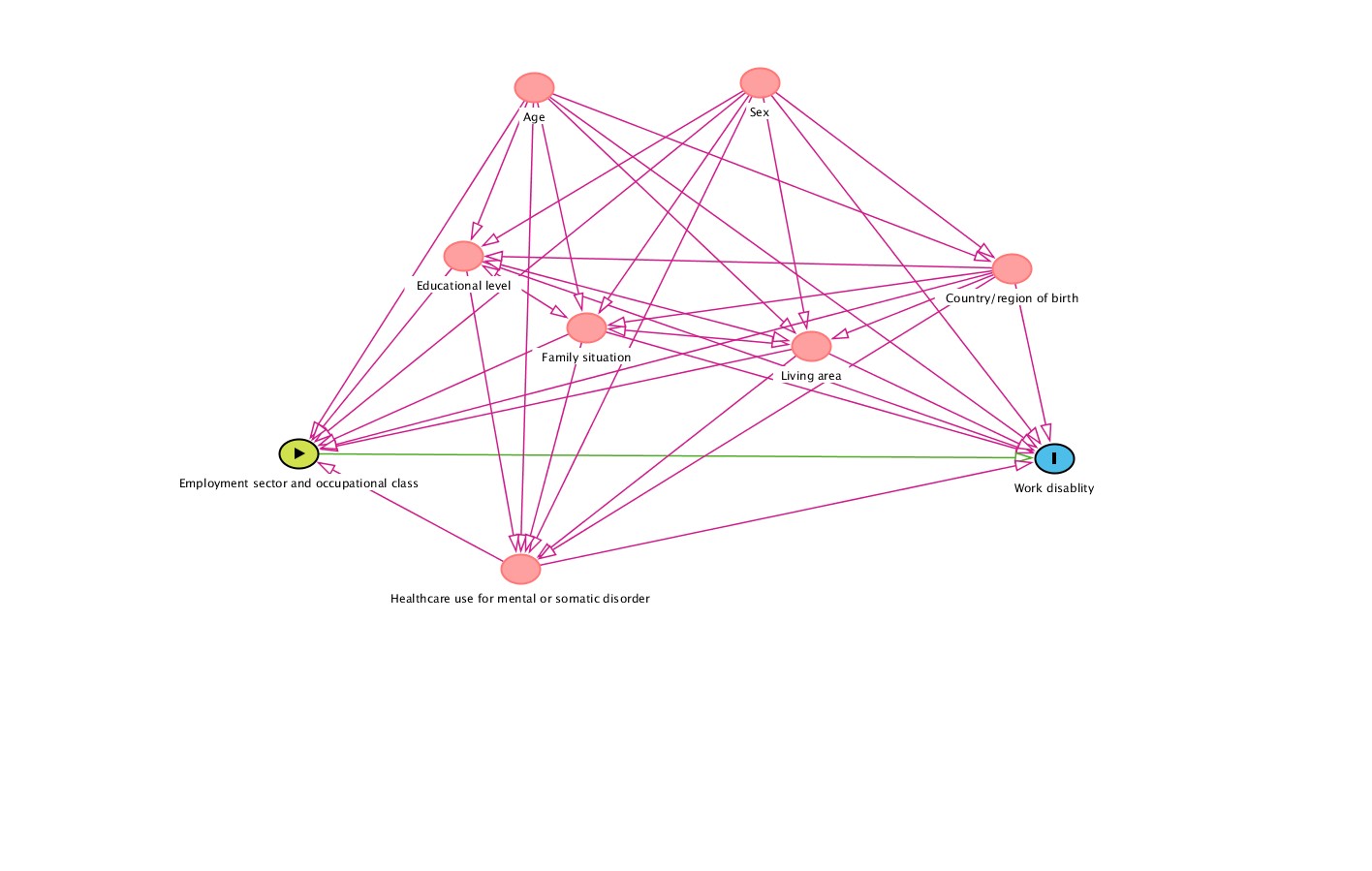

Supplement: ckad026_Supplementary_Data [file ckad026_supplementary_data.docx]
